# Supplementary figures and images for: Transmembrane protein CD69 acts as an S1PR1 agonist
Source: eLife. 2023 Apr 11;12:e88204. doi: 10.7554/eLife.88204 (PMC10154026; doi:10.7554/eLife.88204)

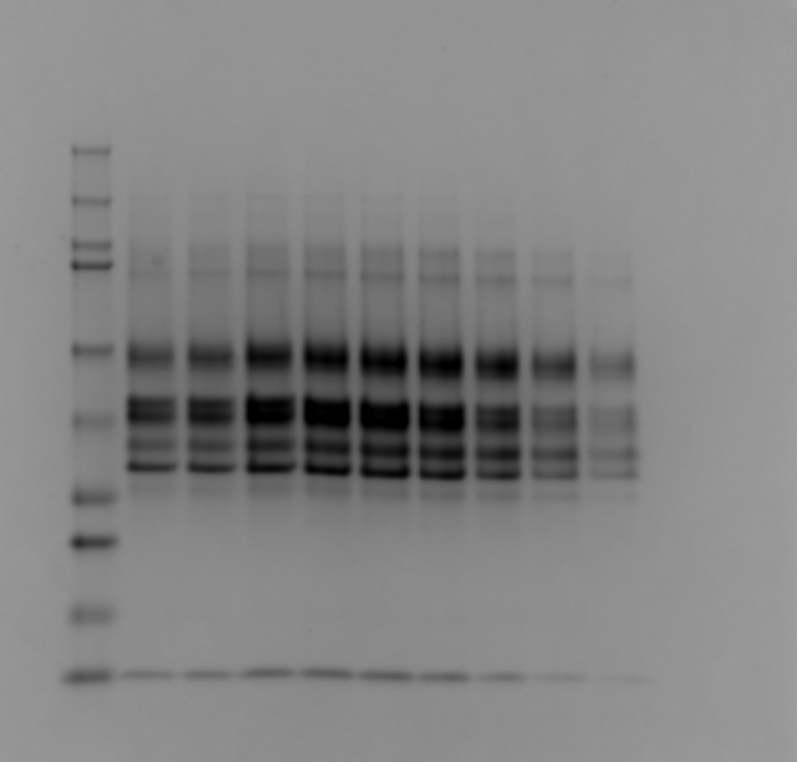

Supplement: Figure 1—figure supplement 1—source data 1. [file elife-88204-fig1-figsupp1-data1.zip › Figure 1ΓÇöfigure supplement 1ΓÇösource data 1/CD69-S1PR1-Gi-scFv16_SDS-PAGE gel for size exclusion chromatography.tif]

kDa

250-  
150-  
75-  
50-  
37-  
25-  
20-  
15-  
10-

— CD69 dimer  
—  $G\alpha_{i1}$   
—  $G\beta$   
— S1PR1  
— scFv16  
—  $G\gamma$

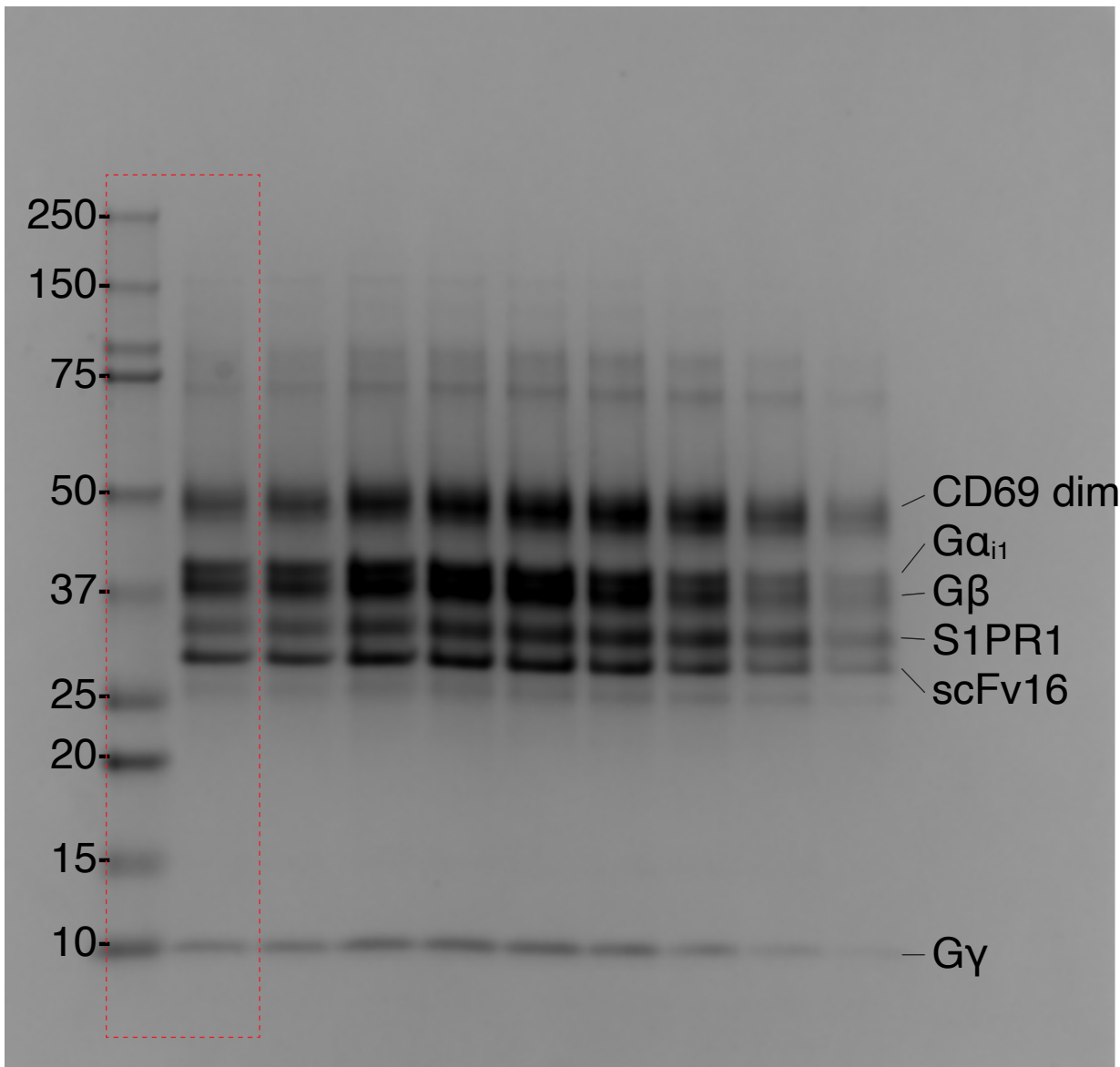

Supplement: Figure 1—figure supplement 1—source data 2. [file elife-88204-fig1-figsupp1-data2.pdf]

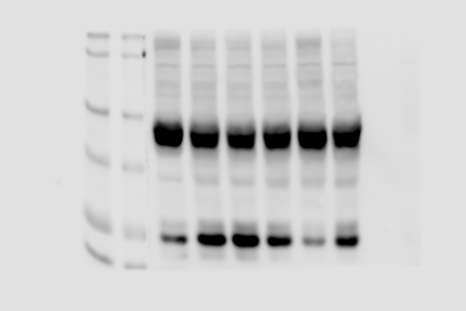

Supplement: Figure 2—source data 1. [file elife-88204-fig2-data1.zip › Figure 2ΓÇösource data 1/Lysate_anti-StrepII-CD69.tif]

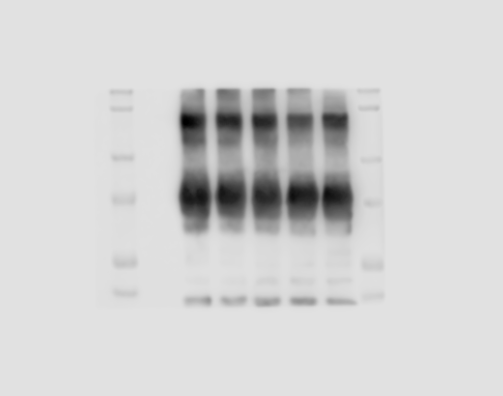

Supplement: Figure 2—source data 1. [file elife-88204-fig2-data1.zip › Figure 2ΓÇösource data 1/IP_anti-FLAG-S1PR1.tif]

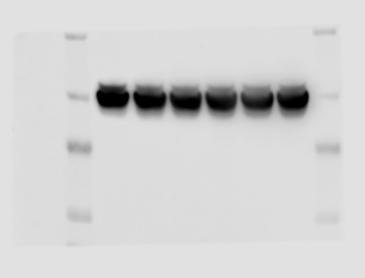

Supplement: Figure 2—source data 1. [file elife-88204-fig2-data1.zip › Figure 2ΓÇösource data 1/Lysate_anti-Tubulin.tif]

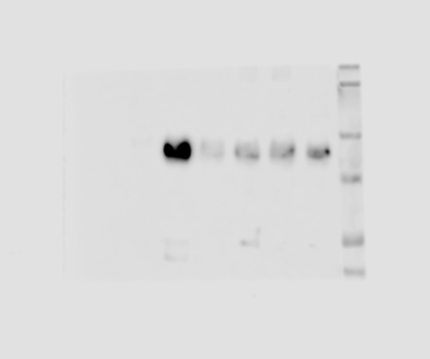

Supplement: Figure 2—source data 1. [file elife-88204-fig2-data1.zip › Figure 2ΓÇösource data 1/IP_anti-StrepII-CD69.tif]

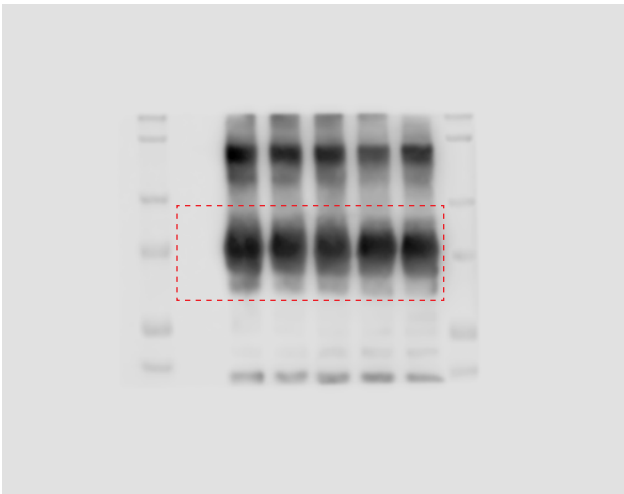

**S1PR1**

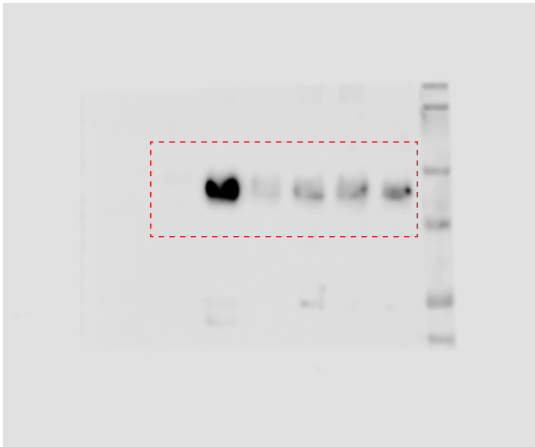

**CD69**

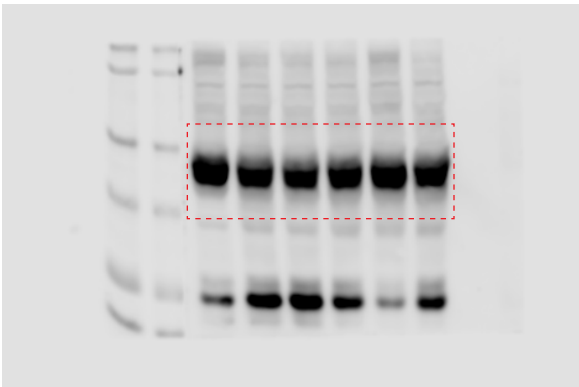

**CD69**

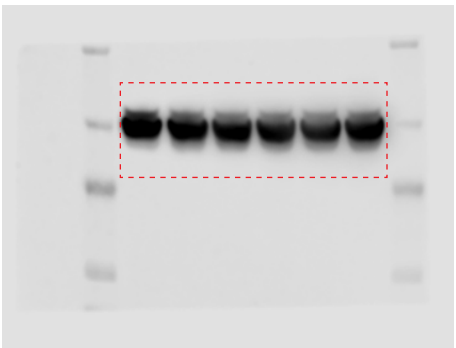

**Tubulin**

Supplement: Figure 2—source data 2. [file elife-88204-fig2-data2.pdf]

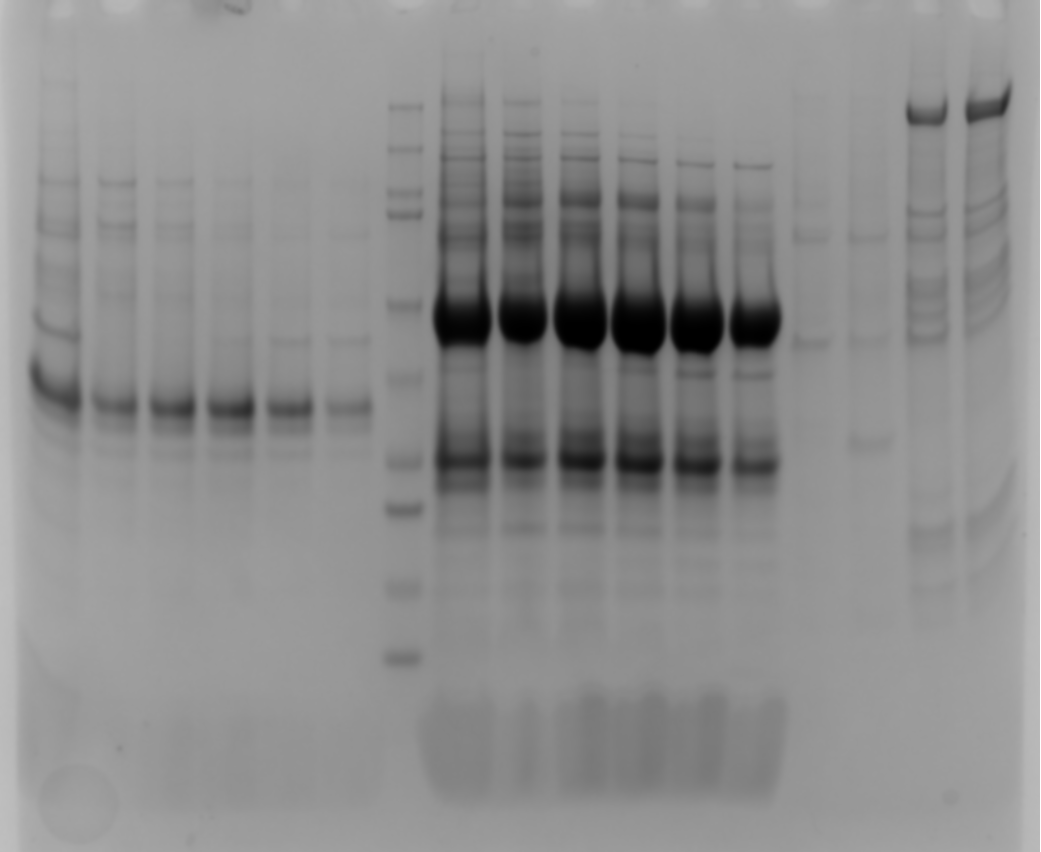

Supplement: Figure 2—figure supplement 1—source data 1. [file elife-88204-fig2-figsupp1-data1.zip › Figure 2ΓÇöfigure supplement 1ΓÇösource data 1/CD69-wild type_SDS-PAGE gel_Coomassie stain.tif]

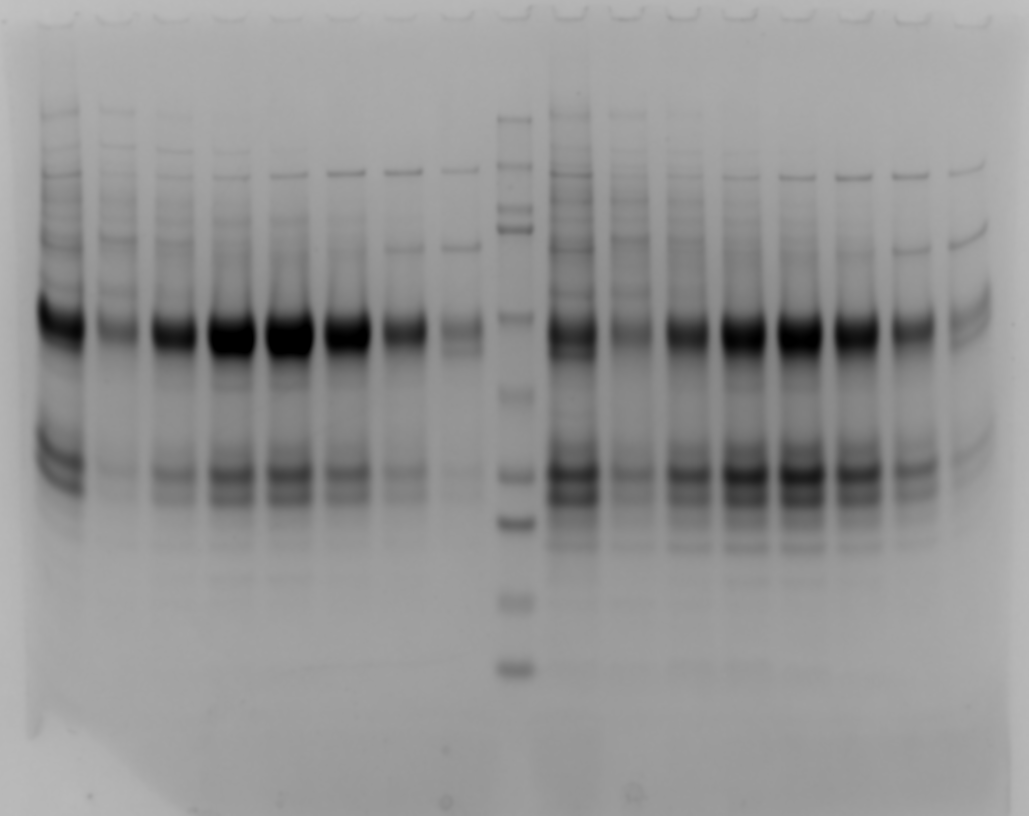

Supplement: Figure 2—figure supplement 1—source data 1. [file elife-88204-fig2-figsupp1-data1.zip › Figure 2ΓÇöfigure supplement 1ΓÇösource data 1/CD69-V48F,V49F and I56F,I59F_SDS-PAGE gel_Coomassie stain.tif]

**CD69 (WT)**

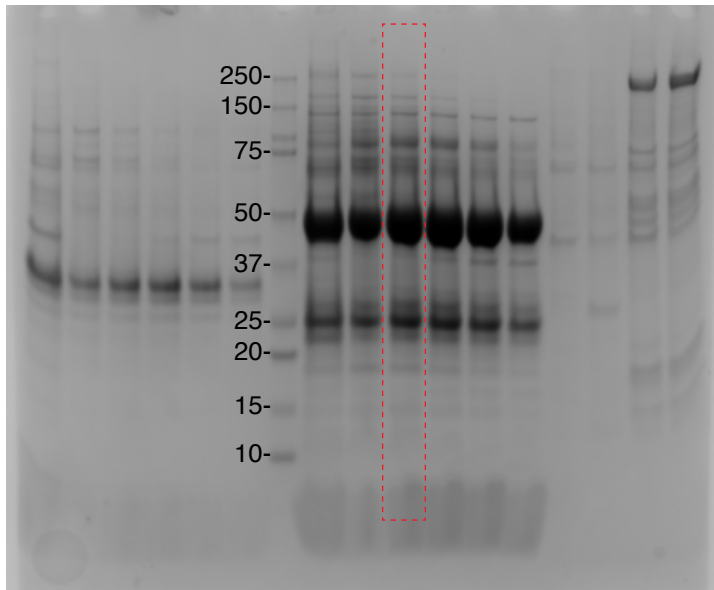

**CD69 (V48F/V49F)**

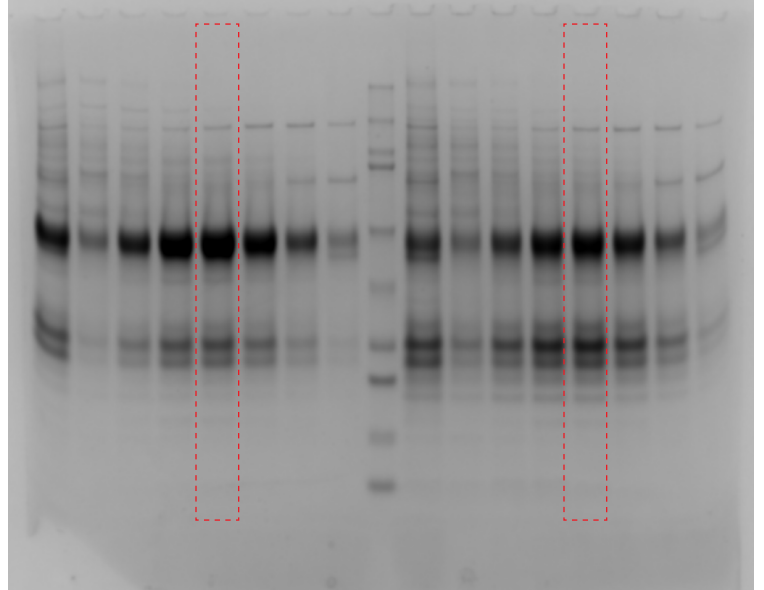

**CD69 (I56F/I59F)**

Supplement: Figure 2—figure supplement 1—source data 2. [file elife-88204-fig2-figsupp1-data2.pdf]

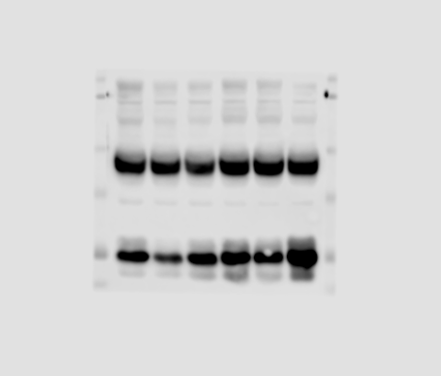

Supplement: Figure 3—figure supplement 1—source data 1. [file elife-88204-fig3-figsupp1-data1.zip › Figure 3ΓÇöfigure supplement 1ΓÇösource data 1/Lysate_anti-StrepII-CD69.tif]

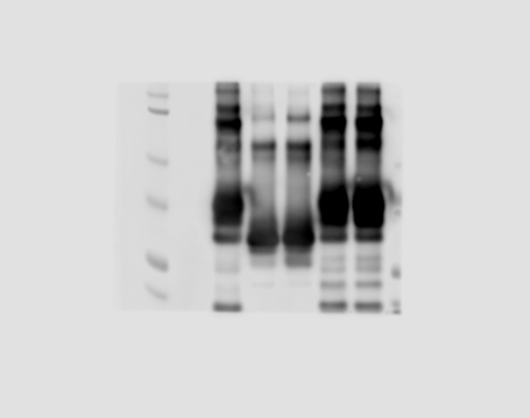

Supplement: Figure 3—figure supplement 1—source data 1. [file elife-88204-fig3-figsupp1-data1.zip › Figure 3ΓÇöfigure supplement 1ΓÇösource data 1/IP_anti-FLAG-S1PR1.tif]

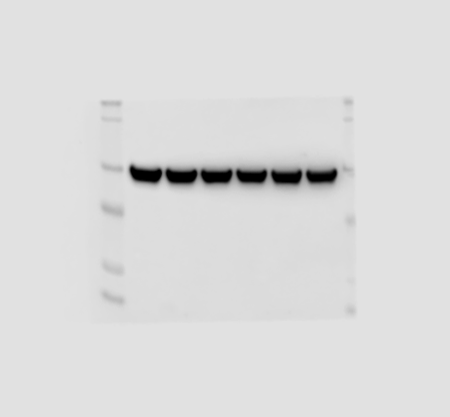

Supplement: Figure 3—figure supplement 1—source data 1. [file elife-88204-fig3-figsupp1-data1.zip › Figure 3ΓÇöfigure supplement 1ΓÇösource data 1/Lysate_anti-Tubulin.tif]

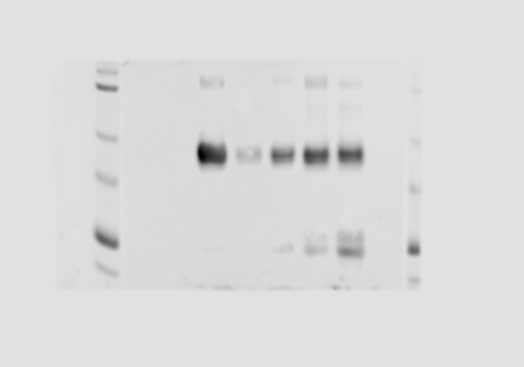

Supplement: Figure 3—figure supplement 1—source data 1. [file elife-88204-fig3-figsupp1-data1.zip › Figure 3ΓÇöfigure supplement 1ΓÇösource data 1/IP_anti-StrepII-CD69.tif]

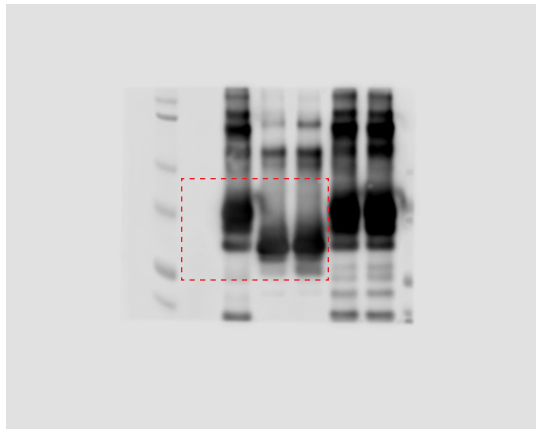

**S1PR1**

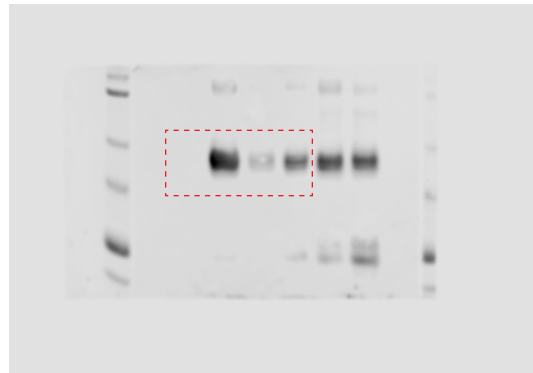

**CD69**

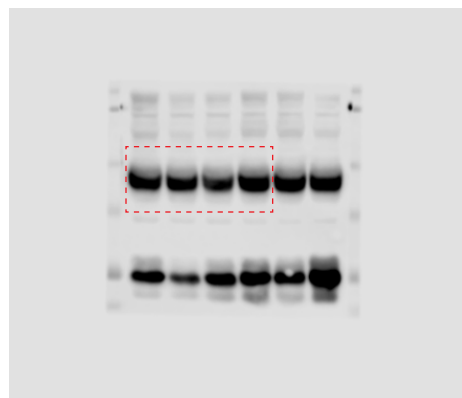

**CD69**

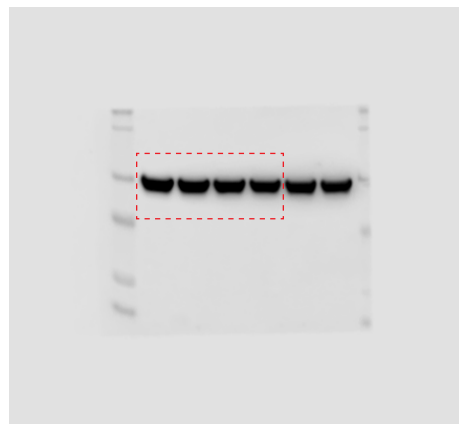

**Tubulin**

Supplement: Figure 3—figure supplement 1—source data 2. [file elife-88204-fig3-figsupp1-data2.pdf]
